# Supplementary material for: Nitrogen Fertilizer Induced Alterations in The Root Proteome of Two Rice Cultivars
Source: Int J Mol Sci. 2019 Jul 26;20(15):3674. doi: 10.3390/ijms20153674 (PMC6695714; doi:10.3390/ijms20153674)
Supplement: Supplementary file 1 [file ijms-20-03674-s001.zip › ijms-531068-for proofreading sup/Figure S3.docx]

**Distribution of peptide fragment ion peak area**

PRM was quantified by peak area. The distribution of fragment ion peak area of the selected peptide in the 12 samples was shown as follows:


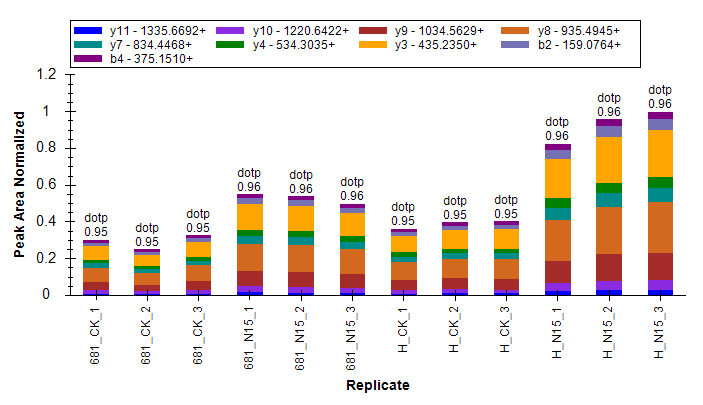


**(1)** The fragment ion peak area distribution of the peptide SATDWVTSNVVPYR (corresponding to protein B8B6Z6) in the 12 samples.


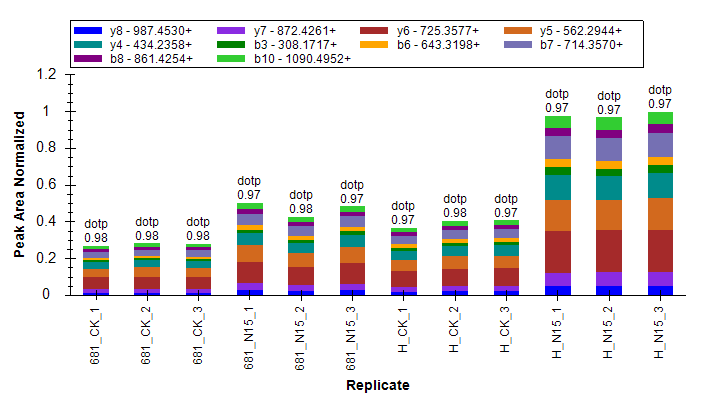


**(2)** The fragment ion peak area distribution of the peptide VAHATYAFNDFYQTTGR (corresponding to protein B8B6Z6) in the 12 samples.


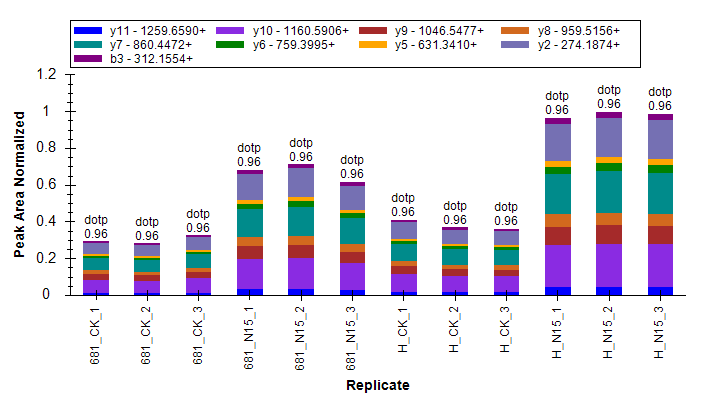


**(3)** The fragment ion peak area distribution of the peptide DVPVNSVTQELDVR (corresponding to protein B8ARU3) in the 12 samples.


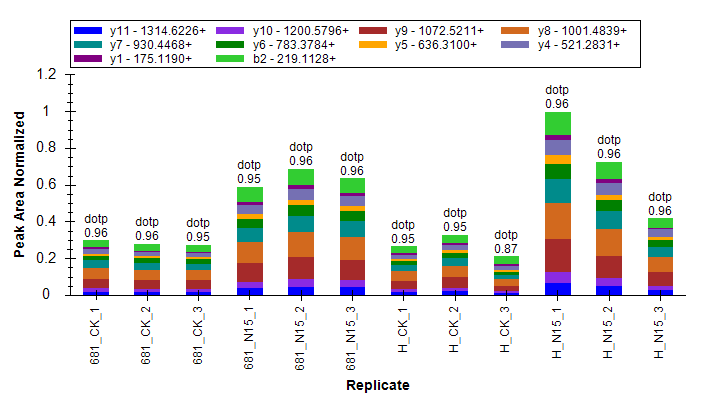


**(4)** The fragment ion peak area distribution of the peptide FALNQAAFFDQFAR (corresponding to protein B8ARU3) in the 12 samples.


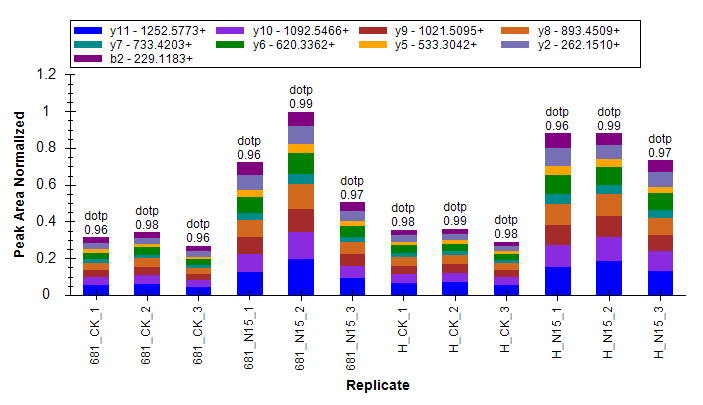


**(5)** The fragment ion peak area distribution of the peptide DLSPLTCAQCLSTAVSR (corresponding to protein A2XF52) in the 12 samples.


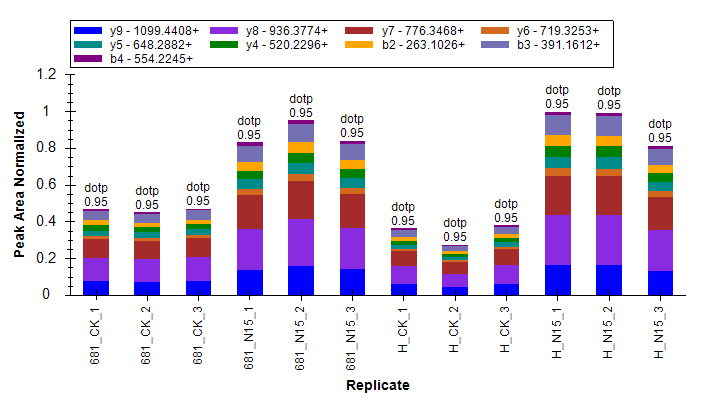


**(6)** The fragment ion peak area distribution of the peptide FDQYCGAQQGCR (corresponding to protein A2XF52) in the 12 samples.


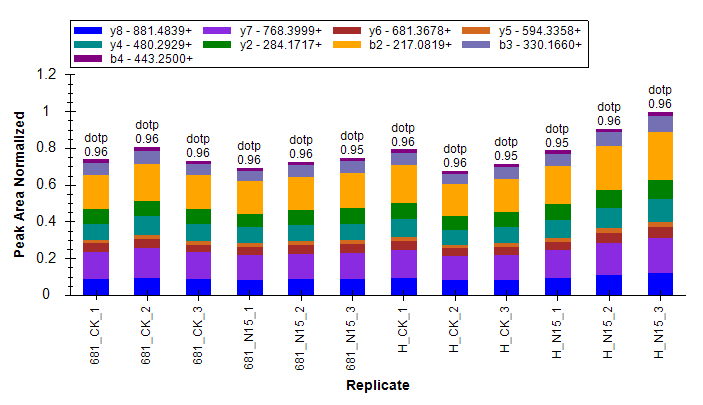


**(7)** The fragment ion peak area distribution of the peptide SELLLSSNPVHK (corresponding to protein A2Z9J9) in the 12 samples.


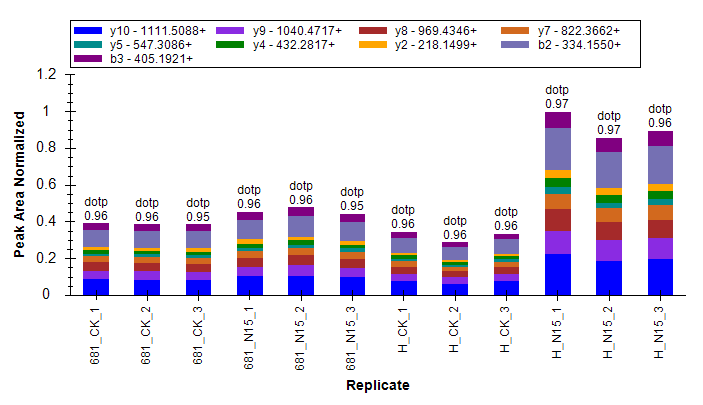


**(8)** The fragment ion peak area distribution of the peptide FWAAFCDDTIAK (corresponding to protein A2Z9J9) in the 12 samples.


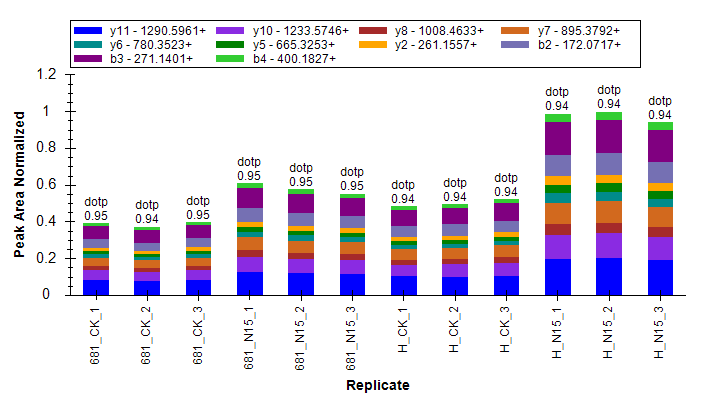


**(9)** The fragment ion peak area distribution of the peptide NGVEQGPQIDDEQFNK (corresponding to protein A2YBK1) in the 12 samples.


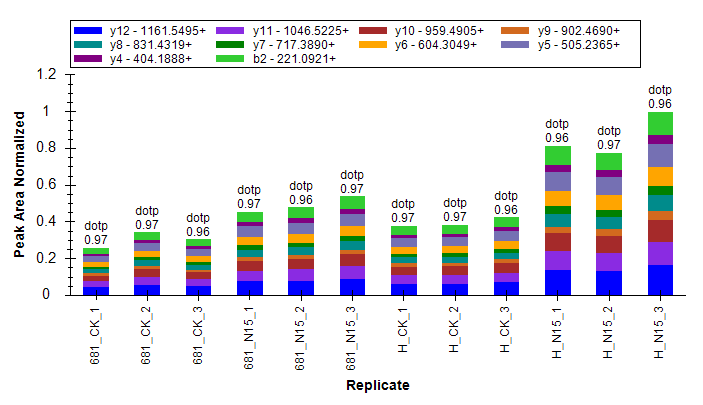


**(10)** The fragment ion peak area distribution of the peptide YGVDSGANLVTGGDR (corresponding to protein A2YBK1) in the 12 samples.


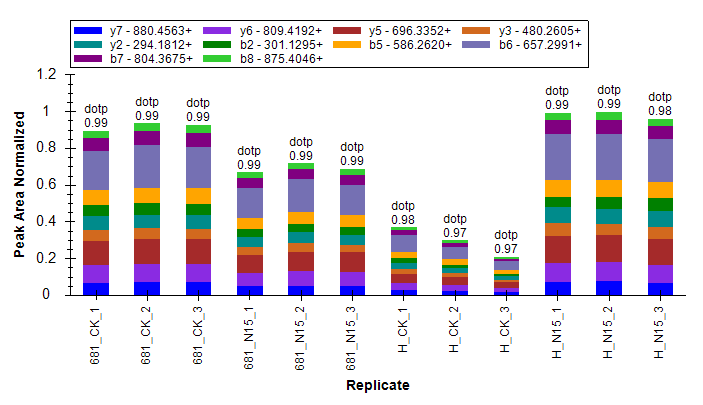


**(11)** The fragment ion peak area distribution of the peptide HYVDAAFALSEWFK (corresponding to protein A2ZDP0) in the 12 samples.


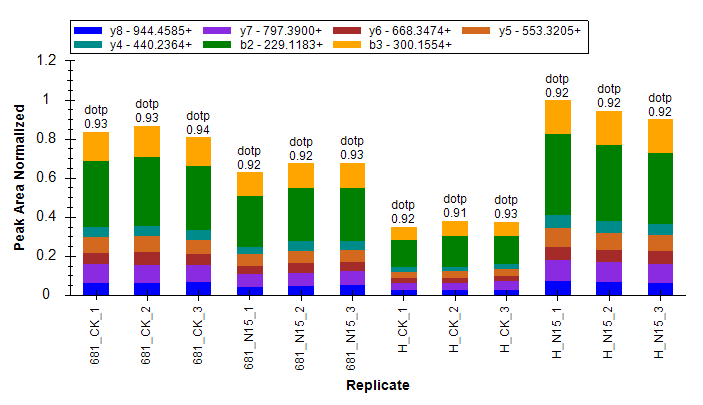


**(12)** The fragment ion peak area distribution of the peptide DLASPVPSPFEDLHGAR (corresponding to protein A2ZDP0) in the 12 samples.


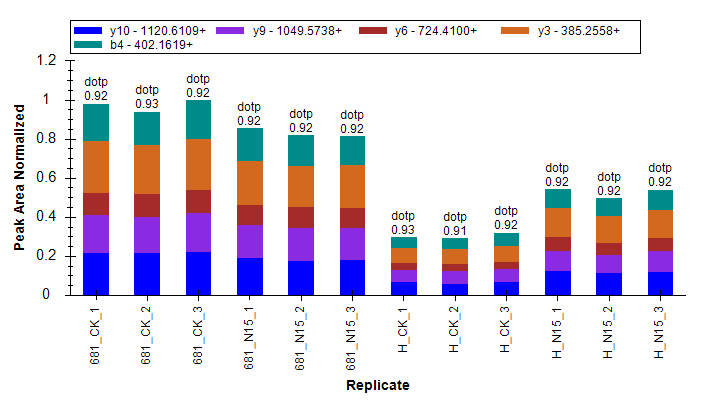


**(13)** The fragment ion peak area distribution of the peptide SAQDIALADLPTTHPIR (corresponding to protein A2XL95) in the 12 samples.


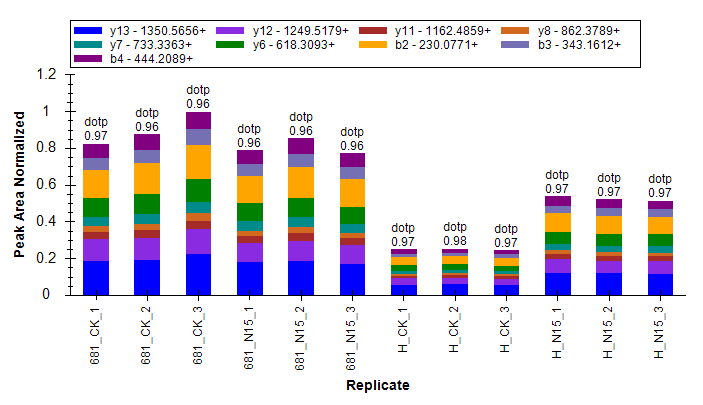


**(14)** The fragment ion peak area distribution of the peptide DNLTLWTSDNAEDGGDEIK (corresponding to protein A2XL95) in the 12 samples.


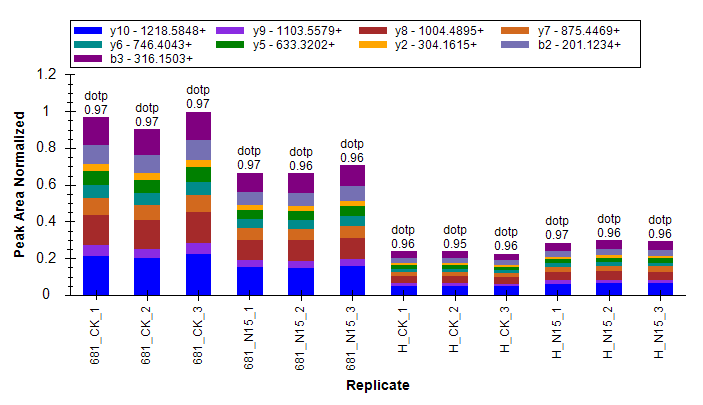


**(15)** The fragment ion peak area distribution of the peptide TVDVEELTVEER (corresponding to protein A2YVG3) in the 12 samples.


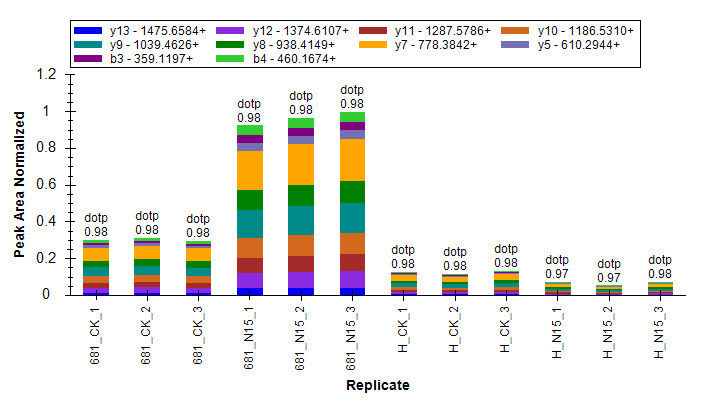


**(16)** The fragment ion peak area distribution of the peptide DDQTSTFTCPAGTNYR (corresponding to protein A2YLZ5) in the 12 samples.


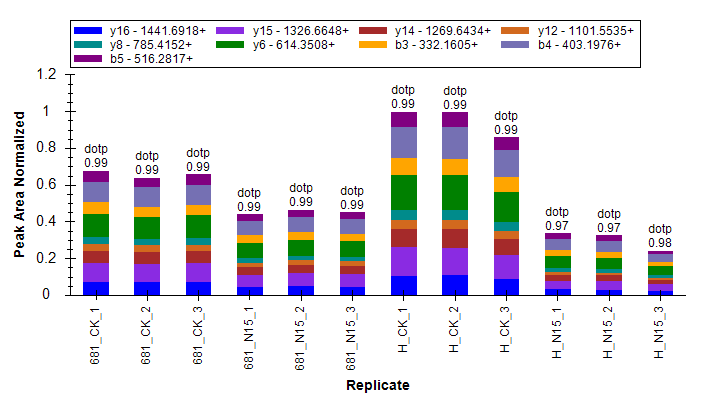


**(17)** The fragment ion peak area distribution of the peptide SPFALADGPASSAAGNPGEIAK (corresponding to protein A2YB91) in the 12 samples.


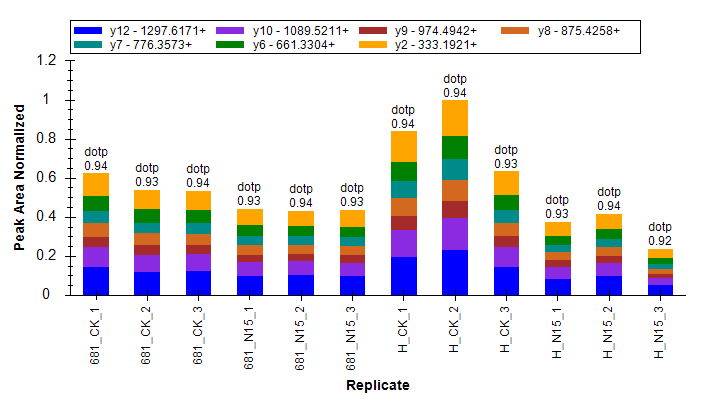


**(18)** The fragment ion peak area distribution of the peptide LIAELNEILAHDVVDEAGAWK (corresponding to protein A2YB91) in the 12 samples.


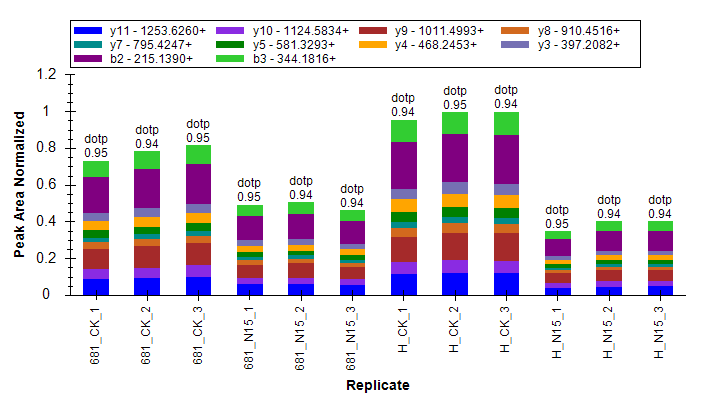


**(19)** The fragment ion peak area distribution of the peptide TIEITDDVLASYK (corresponding to protein B8B9Z2) in the 12 samples.


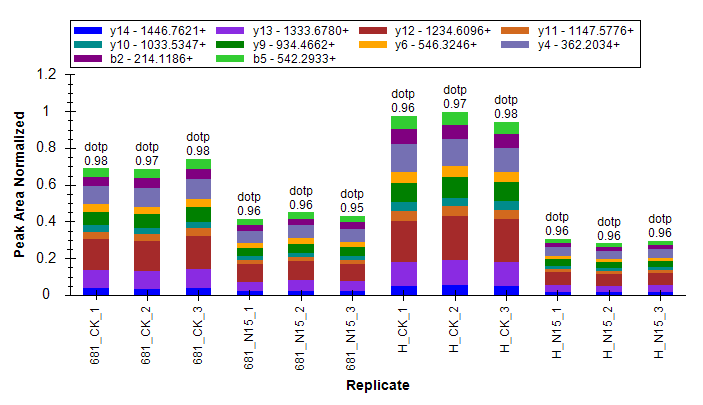


**(20)** The fragment ion peak area distribution of the peptide VNSQLVSNVCLDAISAGK (corresponding to protein B8B9Z2) in the 12 samples.


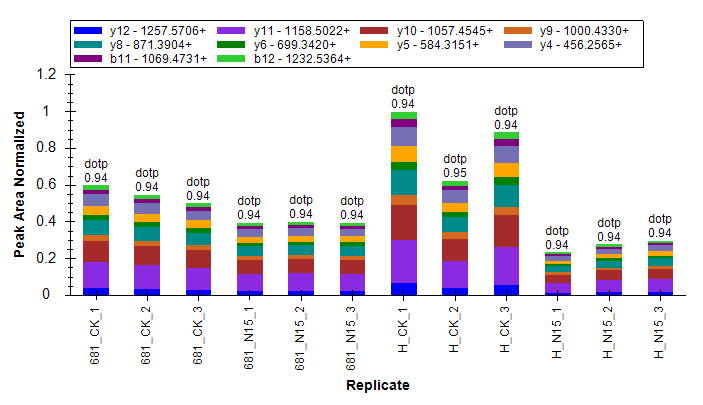


**(21)** The fragment ion peak area distribution of the peptide STPGHTAGCVTYVTGEGDDQPSPR (corresponding to protein B8A774) in the 12 samples.


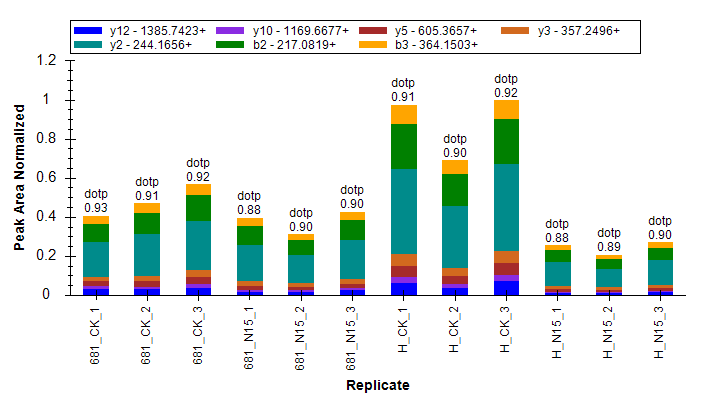


**(22)** The fragment ion peak area distribution of the peptide TDFQGGSSDELYESVHSQIFTLPK (corresponding to protein B8A774) in the 12 samples.


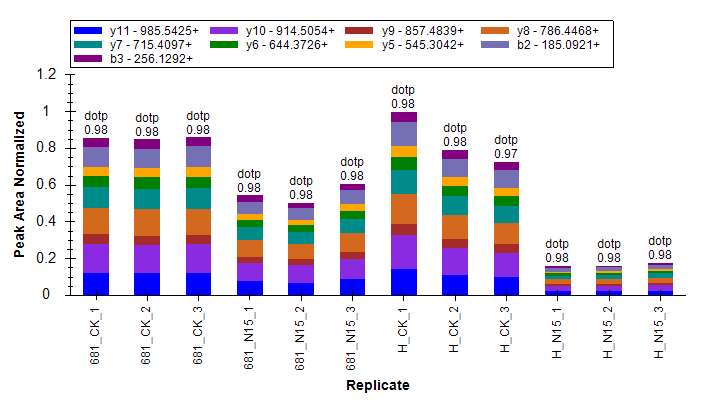


**(23)** The fragment ion peak area distribution of the peptide SPAGAAAVVEAAR (corresponding to protein A2XK19) in the 12 samples.


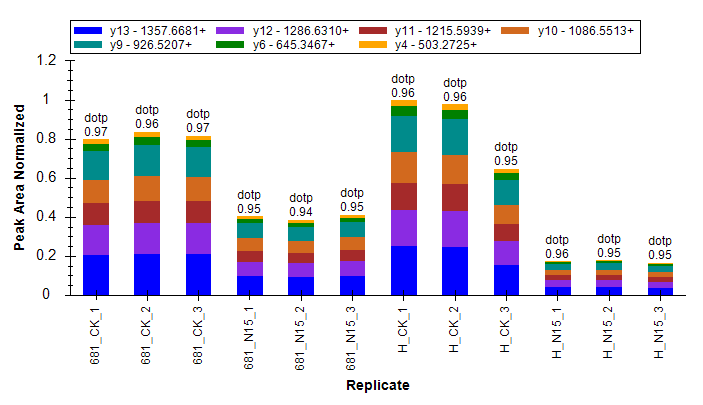


**(24)** The fragment ion peak area distribution of the peptide FGGFSVAAECPALAAWAAR (corresponding to protein A2XK19) in the 12 samples.


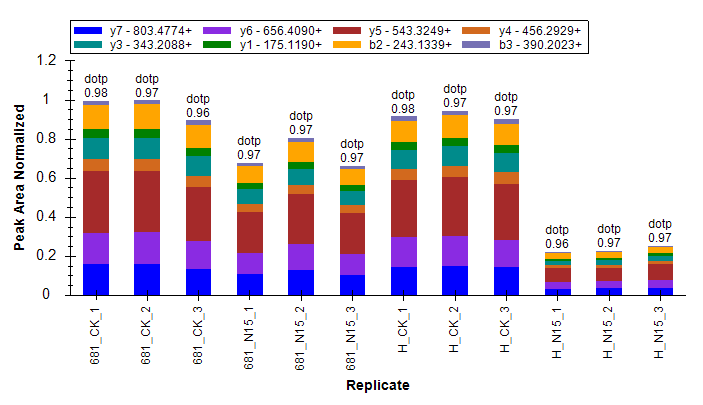


**(25)** The fragment ion peak area distribution of the peptide ELFLSLPAR (corresponding to protein A2Y8B2) in the 12 samples.


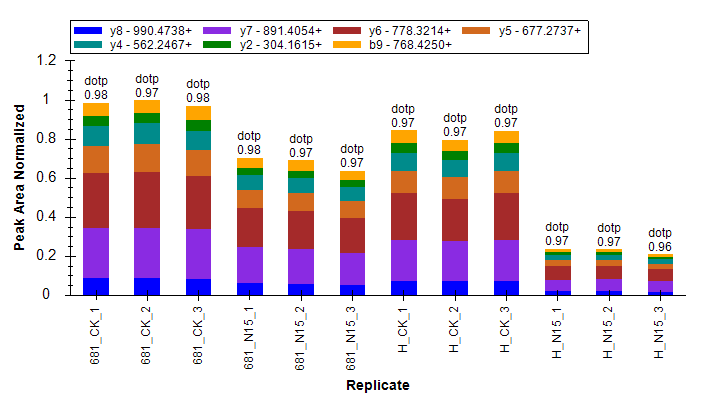


**(26)** The fragment ion peak area distribution of the peptide AGVLGVAAELVLTDEEER (corresponding to protein A2Y8B2) in the 12 samples.


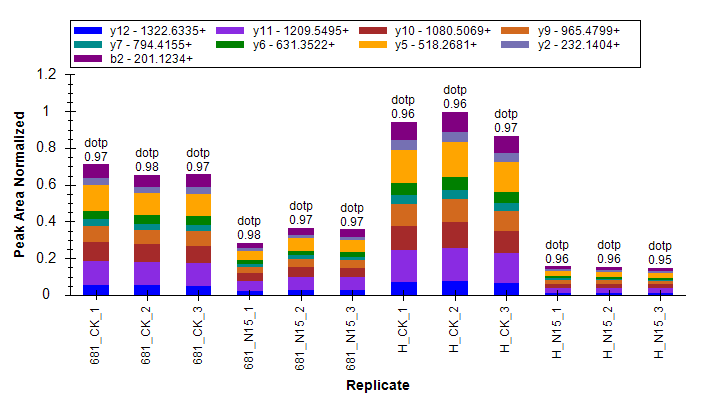


**(27)** The fragment ion peak area distribution of the peptide SLLDSNLEDGNYISAQGR (corresponding to protein B8AWG6) in the 12 samples.


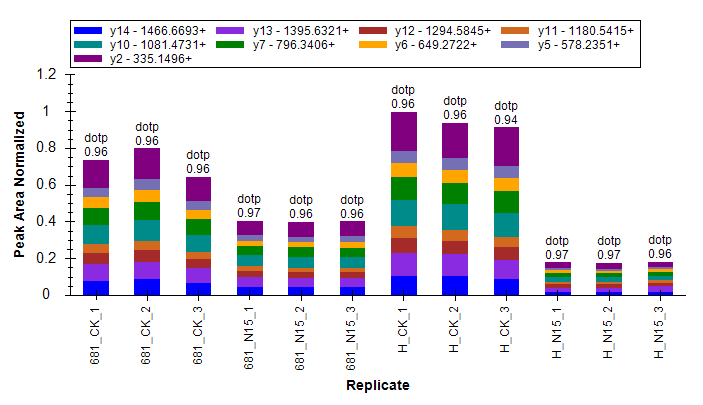


**(28)** The fragment ion peak area distribution of the peptide AQFGNFATNVEGVFAAGDCR (corresponding to protein B8AWG6) in the 12 samples.


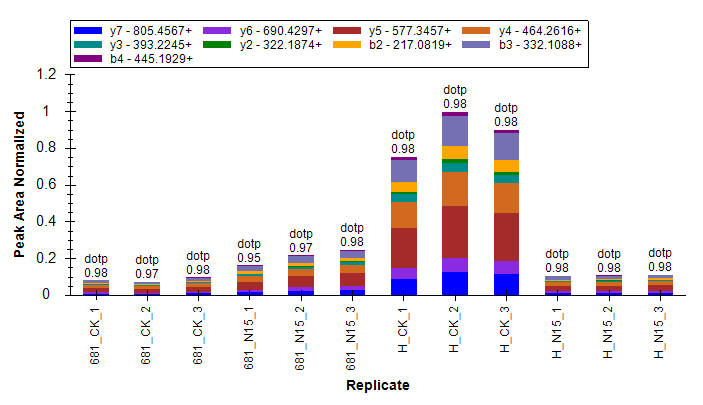


**(29)** The fragment ion peak area distribution of the peptide DTDILAAFR (corresponding to protein P0C511) in the 12 samples.


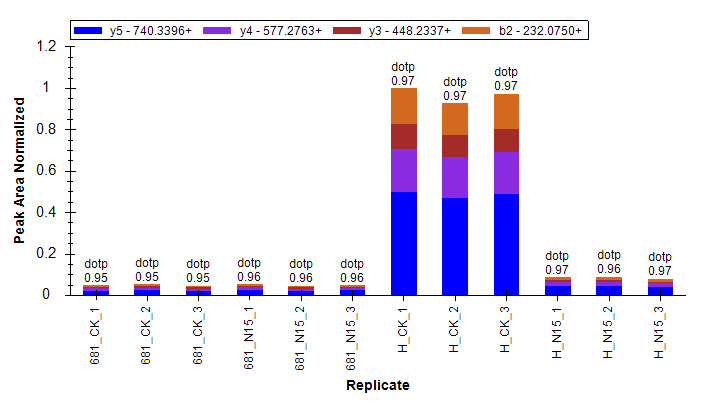


**(30)** The fragment ion peak area distribution of the peptide ACYECLR (corresponding to protein P0C511) in the 12 samples.
