# Supplementary material for: Nitrogen Fertilizer Induced Alterations in The Root Proteome of Two Rice Cultivars
Source: Int J Mol Sci. 2019 Jul 26;20(15):3674. doi: 10.3390/ijms20153674 (PMC6695714; doi:10.3390/ijms20153674)
Supplement: Supplementary file 1 [file ijms-20-03674-s001.zip › ijms-531068-for proofreading sup/Table S3.docx]

**Table S3** The differentially expressed proteins involved in substance metabolism in the comparisons of H_N15 vs H_CK, and 681_N15 vs 681_CK.

| **Protein Accession** | **Protein Description** | **Gene Name** | **H_N15/H_CK** | **681_N15/681_CK** |
| --- | --- | --- | --- | --- |
| Amino acid metabolism | | | | |
| A2XPT4 | amidase 1 | *OsI_14645* | 4.119 | 1.312 |
| B8BET6 | Aminopeptidase | *OsI_31059* | 2.208 | 1.912 |
| A2XYC1 | probable isoaspartyl peptidase/L-asparaginase 2 | *OsI_17700* | 0.965 | 2.076 |
| B8ALL8 | asparagine synthetase [glutamine-hydrolyzing] 1 | *OsI_11112* | 0.955 | 3.581 |
| A2XE78 | Branched-chain-amino-acid aminotransferase | *OsI_10629* | 1.291 | 2.940 |
| B8BGM4 | alanine aminotransferase 2 | *OsI_33417* | 0.400 | 0.731 |
| B8APP5 | alanine aminotransferase 2 isoform X1 | *OsI_10280* | 0.399 | 0.749 |
| B8AZ97 | alanine--glyoxylate aminotransferase 2 homolog 2, mitochondrial | *OsI_20323* | 0.366 | 1.098 |
| A2Z9C9 | methionine gamma-lyase | *OsI_34329* | 1.428 | 3.046 |
| A2ZDN8 | probable O-methyltransferase 2 | *OsI_35904* | 3.299 | 1.583 |
| A2ZCW8 | probable O-methyltransferase 2 | *OsI_35633* | 2.885 | 0.397 |
| A2ZDP0 | probable O-methyltransferase 2 | *OsI_35906* | 2.028 | 0.717 |
| A2ZMI7 | homocysteine S-methyltransferase 2 | *OsI_39039* | 1.741 | 4.209 |
| A2WU38 | aspartic proteinase nepenthesin-1 | *OsI_03384* | 2.229 | 1.230 |
| A2Z9S0 | aspartic proteinase nepenthesin-1-like | *OsI_34483* | 0.472 | 0.949 |
| A2Z9S4 | aspartic proteinase nepenthesin-2-like | *OsI_34487* | 0.309 | 1.070 |
| A2Z5W7 | thiosulfate sulfurtransferase 18 | *OsI_38147* | 1.315 | 3.917 |
| B8AGV2 | thiosulfate sulfurtransferase 18 isoform X1 | *OsI_05463* | 0.459 | 0.619 |
| A2X137 | thiosulfate sulfurtransferase 16, chloroplastic isoform X3 | *OsI_05918* | 0.446 | 0.940 |
| B8BPH1 | sulfite oxidase | *OsI_38220* | 0.338 | 0.725 |
| A2YDH5 | Thionin | *OsI_23164* | 2.450 | 2.324 |
| Carbohydrate Metabolism | | | | |
| A2Z7U4 | Glycosyltransferase | *OsI_33780* | 2.773 | 0.692 |
| A2WUT6 | Glycosyltransferase | *OsI_03644* | 2.178 | 1.230 |
| A2WL58 | galactinol--sucrose galactosyltransferase | *OsI_00571* | 1.344 | 4.351 |
| A2XG08 | Hexosyltransferase | *OsI_11309* | 1.118 | 2.095 |
| A6N0B6 | Ribulose bisphosphate carboxylase small chain | *OsI_38046* | 0.189 | 0.800 |
| P0C511 | Ribulose bisphosphate carboxylase large chain | *rbcL* | 0.173 | 1.293 |
| B8B1F4 | probable alpha-glucosidase | *OsI_24144* | 2.699 | 1.274 |
| B8B1F5 | probable alpha-glucosidase | *OsI_24145* | 2.653 | 1.311 |
| B8B6Z6 | glucan endo-1,3-beta-glucosidase 12 | *OsI_26343* | 2.390 | 1.576 |
| A2WYZ7 | glucan endo-1,3-beta-glucosidase GV | *OsI_05162* | 2.338 | 2.543 |
| A2WYX6 | glucan endo-1,3-beta-glucosidase GII | *OsI_05142* | 2.250 | 2.008 |
| A2WYX5 | glucan endo-1,3-beta-glucosidase, acidic isoform | *OsI_05141* | 2.097 | 1.310 |
| B8AYU2 | "fructose-1,6-bisphosphatase, cytosolic | *OsI_04558* | 1.204 | 2.262 |
| A2Z5S5 | phosphoenolpyruvate carboxykinase [ATP] | *OsI_33003* | 0.376 | 0.604 |
| A2YB91 | Pyrophosphate--fructose 6-phosphate 1-phosphotransferase subunit beta | *PFP-BETA* | 0.459 | 0.653 |
| B8B9Z2 | Pyrophosphate--fructose 6-phosphate 1-phosphotransferase subunit alpha | *PFP-ALPHA* | 0.453 | 0.614 |
| B8B4J4 | Fructose-bisphosphate aldolase | *OsI_23662* | 0.323 | 0.843 |
| A2ZBX1 | Fructose-bisphosphate aldolase | *OsI_35277* | 0.192 | 1.014 |
| A2YGL2 | Xyloglucan endotransglucosylase/hydrolase | *OsI_24318* | 1.078 | 2.442 |
| A2ZF20 | Xyloglucan endotransglucosylase/hydrolase | *OsI_36383* | 0.455 | 0.590 |
| Lipid metabolism | | | | |
| A2XLT7 | Lipoxygenase | *OsI_13441* | 2.774 | 2.015 |
| A2XL11 | Lipoxygenase | *OsI_13155* | 0.482 | 1.023 |
| B8BLY4 | probable carboxylesterase 17 | *OsI_37397* | 2.206 | 1.403 |
| A2WYS7 | Esterase PIR7B | *PIR7B* | 1.670 | 2.606 |

Note: In this present study, proteins with the threshold change fold >2 or <0.5, and *p* value <0.05 were considered as up-regulated and down-regulated proteins, respectively. Black color represented up-regulated protein, and gray color indicated down-regulated protein. N15 represented rice under nitrogen fertilizer treatment with the concentration of 225 kg/hm2, while CK represented the control without N fertilizer; 681 and H represented Quanliangyou 681 and Huanghuazhan cultivar, respectively.
