# Supplementary material for: Nitrogen Fertilizer Induced Alterations in The Root Proteome of Two Rice Cultivars
Source: Int J Mol Sci. 2019 Jul 26;20(15):3674. doi: 10.3390/ijms20153674 (PMC6695714; doi:10.3390/ijms20153674)
Supplement: Supplementary file 1 [file ijms-20-03674-s001.zip › ijms-531068-for proofreading sup/Table S4.docx]

**Table S4** The differentially expressed proteins involved in stress and defense response in the comparisons of H_N15 vs H_CK, and 681_N15 vs 681_CK.

| **Protein Accession** | **Protein Description** | **Gene Name** | **H_N15/H_CK** | **681_N15/681_CK** |
| --- | --- | --- | --- | --- |
| Stress Related Protein | | | | |
| A2Z9M2 | probable glutathione S-transferase GSTU6 | *OsI_34432* | 2.986 | 1.390 |
| A2WZ34 | probable glutathione S-transferase | *OsI_05202* | 2.464 | 1.296 |
| A2Z9L3 | probable glutathione S-transferase GSTU6 | *OsI_34423* | 2.268 | 1.371 |
| A2Z9J9 | probable glutathione S-transferase GSTU6 | *OsI_34399* | 2.127 | 1.090 |
| A2Z6F5 | probable glutathione S-transferase GSTU6 | *OsI_33235* | 2.107 | 1.161 |
| A2XK19 | probable glutathione S-transferase GSTU1 | *OsI_12788* | 0.377 | 0.576 |
| A2WQ51 | glutathione S-transferase 1 | *OsI_01983* | 0.325 | 0.376 |
| A2WZD9 | Peroxidase | *OsI_05317* | 3.307 | 1.576 |
| A2WPA9 | Peroxidase | *OsI_01682* | 2.813 | 3.320 |
| A2Y0P6 | Peroxidase | *OsI_18569* | 2.759 | 1.381 |
| B8ARU3 | Peroxidase | *OsI_18017* | 2.284 | 1.828 |
| A2WPA2 | Peroxidase | *OsI_01676* | 1.901 | 4.470 |
| B8ARU4 | Peroxidase | *OsI_18019* | 1.683 | 2.028 |
| B8AKG0 | peroxidase | *OsI_12177* | 1.632 | 2.265 |
| B8AXN5 | peroxidase 5 | *OsI_18370* | 1.347 | 2.367 |
| A2WNB5 | Peroxidase | *OsI_01340* | 0.466 | 0.914 |
| B8A9K9 | Peroxidase | *OsI_05316* | 0.407 | 0.830 |
| A2XH59 | Peroxidase | *OsI_11734* | 0.335 | 1.048 |
| A2XH51 | Peroxidase | *OsI_11726* | 0.330 | 0.788 |
| B8AQ75 | Peroxidase | *OsI_11727* | 0.328 | 0.755 |
| A2YGK1 | Peroxidase | *OsI_24307* | 0.277 | 0.668 |
| A2YWB7 | anthocyanidin reductase | *OsI_29630* | 3.566 | 5.331 |
| A2Z839 | short-chain type dehydrogenase/reductase | *OsI_33875* | 2.799 | 2.995 |
| B8B3Z4 | putative 12-oxophytodienoate reductase 5 | *OsI_22156* | 1.590 | 2.852 |
| A2XAV7 | cinnamoyl-CoA reductase 1 | *OsI_09373* | 1.166 | 0.448 |
| A2XAV9 | cinnamoyl-CoA reductase 1 isoform X2 | *OsI_09393* | 0.607 | 0.355 |
| B8B311 | long chain acyl-CoA synthetase 4 | *OsI_21752* | 3.132 | 2.267 |
| B8B5G1 | Fatty acyl-CoA reductase | *OsI_25750* | 2.831 | 1.258 |
| A2ZDX4 | Dehydrin Rab16D | *RAB16D* | 0.739 | 41.147 |
| A2YTZ6 | 11 kDa late embryogenesis abundant protein | *OsI_28805* | 1.858 | 16.758 |
| A2Y720 | Late embryogenesis abundant protein 19 | *LEA19* | 1.039 | 16.974 |
| A2WU85 | Group 3 late embryogenesis abundant protein | *Wsi18* | 0.744 | 8.520 |
| Defense related Protein | | | | |
| B8B5L2 | momilactone A synthase-like | *OsI_27228* | 8.193 | 1.351 |
| B8B5L4 | momilactone A synthase-like | *OsI_27231* | 5.823 | 1.236 |
| A2YPN5 | momilactone A synthase-like isoform X1 | *OsI_27236* | 3.413 | 1.705 |
| A2YPP1 | momilactone A synthase-like | *OsI_27242* | 2.620 | 1.347 |
| A2Z1W4 | momilactone A synthase-like isoform X2 | *OsI_31597* | 2.190 | 1.846 |
| B8B5L1 | momilactone A synthase-like | *OsI_27226* | 2.118 | 1.000 |
| A2Y4F6 | chitinase 9 | *OsI_19884* | 5.058 | 0.787 |
| A2Z7A3 | chitinase 1 | *OsI_33579* | 4.714 | 1.641 |
| A2Y4F5 | chitinase 2 | *OsI_19883* | 2.622 | 1.693 |
| A2WY68 | glycine-rich cell wall structural protein 1.8 | *OsI_04875* | 2.016 | 1.646 |

Note: In this present study, proteins with the threshold change fold >2 or <0.5, and *p* value <0.05 were considered as up-regulated and down-regulated proteins, respectively. Black color represented up-regulated protein, and gray color indicated down-regulated protein. N15 represented rice under nitrogen fertilizer treatment with the concentration of 225 kg/hm2, while CK represented the control without N fertilizer; 681 and H represented Quanliangyou 681 and Huanghuazhan cultivar, respectively.
