# Supplementary material for: Nitrogen Fertilizer Induced Alterations in The Root Proteome of Two Rice Cultivars
Source: Int J Mol Sci. 2019 Jul 26;20(15):3674. doi: 10.3390/ijms20153674 (PMC6695714; doi:10.3390/ijms20153674)
Supplement: Supplementary file 1 [file ijms-20-03674-s001.zip › ijms-531068-for proofreading sup/Table S5.docx]

**Table S5** The differentially expressed proteins in the comparisons of H_CK vs 681_CK, and H_N15 vs 681_N15, involved in ammonium assimilation, signal transduction, energy metabolism, material transport, substance metabolism, and stress/defense response.

| **Protein Accession** | **Protein Description** | **Gene Name** | **H_CK/ 681_CK** | **H_N15/681_N15** |
| --- | --- | --- | --- | --- |
| Ammonium Assimilation | | | | |
| B8AQE3 | Glutamine synthetase | *OsI_13264* | 0.702 | 0.297 |
| B8AZE9 | Glutathione synthetase | *OsI_18978* | 1.652 | 2.120 |
| A2WUY4 | probable glutamate carboxypeptidase 2 | *OsI_03696* | 0.498 | 0.960 |
| Signal Transduction | | | | |
| A2Z8U1 | putative receptor-like protein kinase | *OsI_34134* | 0.772 | 2.269 |
| A2XBX8 | probable receptor-like protein kinase At5g24010 | *OsI_09795* | 0.465 | 0.756 |
| B8ANZ8 | vacuolar-sorting receptor 1 | *OsI_11454* | 0.376 | 0.579 |
| A2Z686 | vacuolar-sorting receptor 3 | *OsI_33166* | 0.467 | 0.605 |
| A2XEP1 | phosphoenolpyruvate carboxykinase [ATP] | *OsI_10803* | 0.937 | 0.495 |
| A2XL95 | 14-3-3-like protein GF14-F isoform X2 | *OsI_13240* | 0.327 | 0.728 |
| A2XUA6 | 14-3-3-like protein GF14-B | *OsI_16185* | 0.266 | 0.676 |
| A2X6G8 | 14-3-3-like protein GF14-E isoform X1 | *OsI_07806* | 0.221 | 0.725 |
| A2YWB4 | 14-3-3-like protein GF14-A | *OsI_29626* | 0.283 | 0.678 |
| A2YVG3 | 14-3-3-like protein GF14-C | *OsI_29320* | 0.341 | 0.576 |
| Energy Metabolism | | | | |
| A2Z4G9 | probable NAD(P)H-dependent oxidoreductase 1 | *OsI_32550* | 1.771 | 3.476 |
| A2XNS0 | Ferredoxin | *OsI_15810* | 0.249 | 1.033 |
| B8AYQ7 | ferredoxin-6, chloroplastic | *OsI_20134* | 0.498 | 0.774 |
| A2ZCK1 | Alcohol dehydrogenase family-3 | *OSI9Ba083O10_092B13-3* | 0.681 | 2.272 |
| B8ADR5 | cytokinin dehydrogenase 2 | *OsI_00771* | 0.771 | 2.055 |
| A2Y7Q3 | Isocitrate dehydrogenase [NADP] | *OsI_21072* | 0.175 | 0.146 |
| A2WS18 | aldehyde dehydrogenase family 2 member C4 | *OsI_02656* | 2.421 | 1.326 |
| A2Y8B2 | L-lactate dehydrogenase | *OsI_21291* | 0.666 | 0.434 |
| A2XU83 | Glyceraldehyde-3-phosphate dehydrogenase | *OsI_16160* | 4.119 | 1.355 |
| Material Transport | | | | |
| B8BAI4 | ABC transporter A family member 7 | *OsI_29140* | 0.548 | 0.465 |
| A2XAP0 | pyrophosphate-energized vacuolar membrane proton pump | *OsI_09320* | 0.700 | 0.399 |
| A2WKJ2 | protein NRT1/ PTR FAMILY 8.2 isoform X2 | *OsI_00345* | 0.737 | 0.452 |
| A2YFN7 | Magnesium transporter MRS2-B | *MRS2-B* | 0.454 | 0.791 |
| A2Z1P3 | copper transporter 5.1 | *OsI_31527* | 0.138 | 0.190 |
| A2YVV8 | putative copper transporter 5.2 | *OsI_29464* | 0.755 | 0.401 |
| A2YML7 | sugar carrier protein C | *OsI_26468* | 0.782 | 0.420 |
| B8B590 | endoplasmic reticulum metallopeptidase 1 | *OsI_25697* | 0.460 | 0.683 |
| B8ACJ8 | calmodulin-like | *OsI_01318* | 0.395 | 0.097 |
| B8B8L5 | probable calcium-binding protein CML13 | *OsI_26889* | 0.465 | 0.616 |
| Amino Acid Metabolism | | | | |
| A2WLW7 | peptide-N4-(N-acetyl-beta-glucosaminyl)asparagine amidase A-like | *OsI_00835* | 0.473 | 0.814 |
| B8AUL1 | subtilisin-like protease SBT3.8 isoform X2 | *OsI_14683* | 0.153 | 0.126 |
| A2Y926 | Proteasome subunit beta type | *OsI_21564* | 0.498 | 0.587 |
| A2XE78 | Branched-chain-amino-acid aminotransferase | *OsI_10629* | 0.916 | 0.402 |
| B8AZ97 | alanine--glyoxylate aminotransferase 2 homolog 2, mitochondrial | *OsI_20323* | 1.187 | 0.395 |
| A2Z9C9 | methionine gamma-lyase | *OsI_34329* | 1.041 | 0.488 |
| A2Y7Y3 | Carboxypeptidase | *OsI_21149* | 0.319 | 0.631 |
| B8B3P4 | serine carboxypeptidase-like 2 | *OsI_22022* | 0.291 | 0.274 |
| A2ZCW8 | probable O-methyltransferase 2 | *OsI_35633* | 0.393 | 2.855 |
| A2ZDP0 | probable O-methyltransferase 2 | *OsI_35906* | 0.381 | 1.079 |
| B8BP68 | O-methyltransferase ZRP4 | *OsI_38209* | 0.832 | 2.030 |
| A2ZMI7 | homocysteine S-methyltransferase 2 | *OsI_39039* | 1.151 | 0.476 |
| A2Z9S7 | aspartic proteinase nepenthesin-1-like | *OsI_34489* | 0.442 | 0.379 |
| A2YM47 | aspartic proteinase nepenthesin-1 | *OsI_26300* | 0.643 | 0.410 |
| A2Z9S3 | aspartic proteinase nepenthesin-1-like | *OsI_34486* | 0.423 | 0.501 |
| A2YM54 | aspartic proteinase nepenthesin-2-like | *OsI_26307* | 0.556 | 0.316 |
| A2XG17 | aspartic proteinase nepenthesin-1-like | *OsI_11320* | 0.480 | 0.453 |
| B8BI17 | aspartic proteinase nepenthesin-1-like | *OsI_34481* | 0.583 | 0.376 |
| A2Z9S4 | aspartic proteinase nepenthesin-2-like | *OsI_34487* | 0.642 | 0.186 |
| A2Z5W7 | thiosulfate sulfurtransferase 18 | *OsI_38147* | 1.343 | 0.451 |
| B8BML3 | metallothionein-like protein 4C | *OsI_38777* | 0.232 | 0.292 |
| Carbohydrate Metabolism | | | | |
| A2Z7U4 | Glycosyltransferase | *OsI_33780* | 0.586 | 2.345 |
| A2WUT6 | Glycosyltransferase | *OsI_03644* | 1.031 | 1.826 |
| B8BMI2 | Glycosyltransferase | *OsI_38729* | 1.934 | 2.204 |
| A2WT11 | Glycosyltransferase | *OsI_03002* | 0.616 | 0.463 |
| A2YZY5 | Glycosyltransferase | *OsI_30917* | 0.173 | 0.233 |
| A2WL58 | galactinol--sucrose galactosyltransferase | *OsI_00571* | 1.084 | 0.335 |
| A6N0B6 | Ribulose bisphosphate carboxylase small chain | *OsI_38046* | 4.712 | 1.112 |
| P0C511 | Ribulose bisphosphate carboxylase large chain | *rbcL* | 6.073 | 0.811 |
| A2WYX5 | glucan endo-1,3-beta-glucosidase, acidic isoform | *OsI_05141* | 1.311 | 2.098 |
| B8B6Z9 | glucan endo-1,3-beta-glucosidase GII isoform X2 | *OsI_26347* | 1.533 | 2.128 |
| A2YM90 | "glucan endo-1,3-beta-glucosidase 7 | *OsI_26344* | 3.348 | 5.752 |
| A2YIJ5 | "glucan endo-1,3-beta-glucosidase 8 | *OsI_25042* | 0.384 | 0.666 |
| B8B6Z4 | "glucan endo-1,3-beta-glucosidase 3 | *OsI_26341* | 3.692 | 5.017 |
| B8B5H9 | alpha-glucosidase 2 isoform X1 | *OsI_25777* | 0.270 | 0.292 |
| B8B5H8 | alpha-glucosidase 2 isoform X2 | *OsI_25776* | 0.308 | 0.327 |
| B8B4J4 | Fructose-bisphosphate aldolase | *OsI_23662* | 2.843 | 1.088 |
| A2ZBX1 | Fructose-bisphosphate aldolase | *OsI_35277* | 4.496 | 0.850 |
| A2YGL2 | Xyloglucan endotransglucosylase/hydrolase | *OsI_24318* | 1.057 | 0.467 |
| A2X3G4 | Xyloglucan endotransglucosylase/hydrolase | *OsI_06752* | 0.221 | 0.376 |
| Lipid Metabolism | | | | |
| A2XL11 | Lipoxygenase | *OsI_13155* | 2.198 | 1.036 |
| A2WYS7 | Esterase PIR7B | *PIR7B* | 2.079 | 1.332 |
| A2X6L9 | glycerophosphodiester phosphodiesterase GDPDL3 | *OsI_07859* | 0.491 | 0.467 |
| A2YH44 | GDSL esterase/lipase | *OsI_24508* | 0.626 | 0.494 |
| Stress Related Protein | | | | |
| A2Z6F5 | probable glutathione S-transferase GSTU6 | *OsI_33235* | 1.200 | 2.178 |
| A2XK19 | probable glutathione S-transferase GSTU1 | *OsI_12788* | 0.761 | 0.499 |
| A2WPA9 | Peroxidase | *OsI_01682* | 0.273 | 0.231 |
| A2WPA2 | Peroxidase | *OsI_01676* | 0.262 | 0.111 |
| B8A9K9 | Peroxidase | *OsI_05316* | 0.722 | 0.354 |
| A2XH51 | Peroxidase | *OsI_11726* | 1.086 | 0.455 |
| A2YGK1 | Peroxidase | *OsI_24307* | 1.108 | 0.460 |
| B8B5G1 | Fatty acyl-CoA reductase | *OsI_25750* | 1.315 | 2.960 |
| A2X854 | dehydrin COR410 | *OsI_08410* | 0.789 | 0.470 |
| A2ZDX4 | Dehydrin Rab16D | *RAB16D* | 1.912 | 0.034 |
| A2YTZ6 | 11 kDa late embryogenesis abundant protein | *OsI_28805* | 1.022 | 0.113 |
| A2Y720 | Late embryogenesis abundant protein 19 | *LEA19* | 1.061 | 0.065 |
| A2WU85 | Group 3 late embryogenesis abundant protein | *Wsi18* | 1.302 | 0.114 |
| Denfense Related Protein | | | | |
| B8B5L2 | momilactone A synthase-like | *OsI_27228* | 1.076 | 6.525 |
| B8B5L4 | momilactone A synthase-like | *OsI_27231* | 0.615 | 2.899 |
| A2YPN5 | momilactone A synthase-like isoform X1 | *OsI_27236* | 0.326 | 0.652 |
| B8B5L1 | momilactone A synthase-like | *OsI_27226* | 0.340 | 0.720 |
| B8B5L3 | momilactone A synthase-like | *OsI_27229* | 0.593 | 0.449 |
| A2Y4F6 | chitinase 9 | *OsI_19884* | 0.289 | 1.858 |
| A2WY68 | glycine-rich cell wall structural protein 1.8 | *OsI_04875* | 0.433 | 0.530 |
| A2X6J9 | glycine-rich cell wall structural protein | *OsI_07842* | 0.836 | 0.408 |
| A2XMV8 | leucine-rich repeat extensin-like protein 6 | *OsI_13883* | 0.480 | 0.411 |
| B8B9H9 | putative disease resistance RPP13-like protein 3 | *OsI_28706* | 0.176 | 0.195 |
| B8B9H8 | putative disease resistance RPP13-like protein 3 | *OsI_28705* | 0.220 | 0.202 |

Note: In this present study, proteins with the threshold change fold >2 or <0.5, and *p* value <0.05 were considered as up-regulated and down-regulated proteins, respectively. Black color represented up-regulated protein, and gray color indicated down-regulated protein. N15 represented rice under nitrogen fertilizer treatment with the concentration of 225 kg/hm2, while CK represented the control without N fertilizer; 681 and H represented Quanliangyou 681 and Huanghuazhan cultivar, respectively.
