# Supplementary figures and images for: Nitrogen Fertilizer Induced Alterations in The Root Proteome of Two Rice Cultivars
Source: Int J Mol Sci. 2019 Jul 26;20(15):3674. doi: 10.3390/ijms20153674 (PMC6695714; doi:10.3390/ijms20153674)

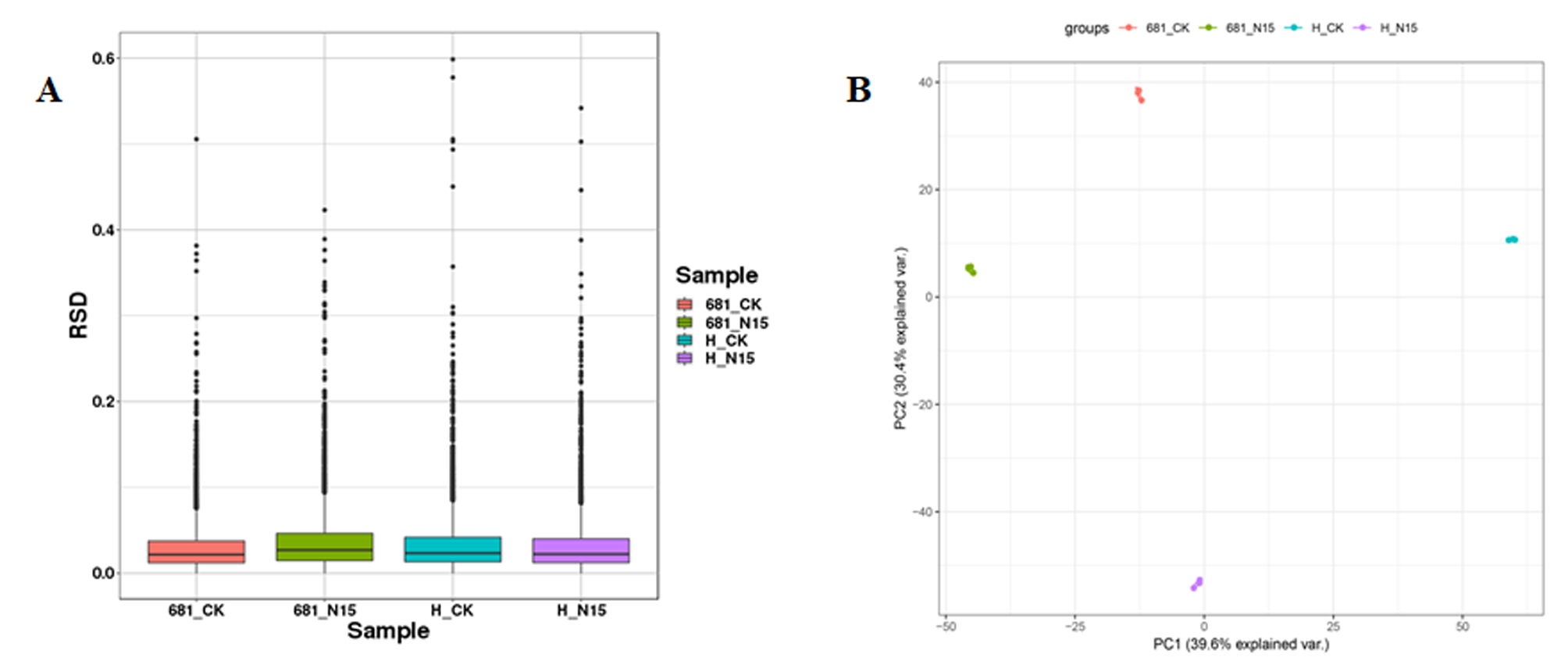

Supplement: Supplementary file 1 [file ijms-20-03674-s001.zip › ijms-531068-for proofreading sup/Figure S1.tif]

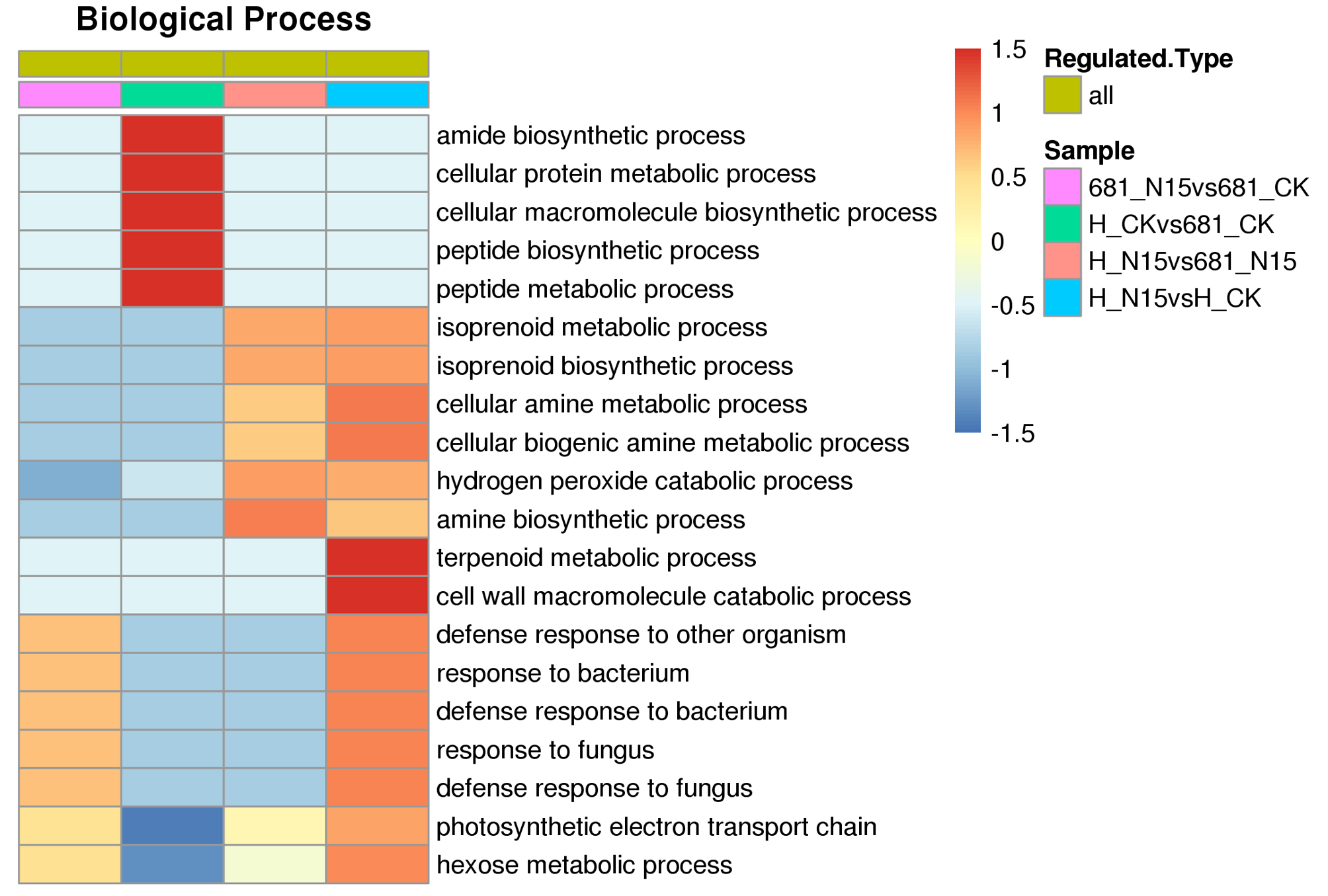

Supplement: Supplementary file 1 [file ijms-20-03674-s001.zip › ijms-531068-for proofreading sup/Figure S2.tif]
